# Analysis of anti-malarial resistance markers in *pfmdr1* and *pfcrt* across Southeast Asia in the Tracking Resistance to Artemisinin Collaboration

**DOI:** 10.1186/s12936-016-1598-6

**Published:** 2016-11-08

**Authors:** Krongkan Srimuang, Olivo Miotto, Pharath Lim, Rick M. Fairhurst, Dominic P. Kwiatkowski, Charles J. Woodrow, Mallika Imwong, for the Tracking Resistance to Artemisinin Collaboration

**Affiliations:** 1 Department of Molecular Tropical Medicine and Genetics, Faculty of Tropical Medicine, Mahidol University, Bangkok, Thailand; 2 Mahidol Oxford Tropical Medicine Research Unit, Bangkok, Thailand; 3 Wellcome Trust Sanger Institute, Hinxton, UK; 4 Medical Research Council (MRC) Centre for Genomics and Global Health, University of Oxford, Oxford, UK; 5 Laboratory of Malaria and Vector Research, National Institute of Allergy and Infectious Diseases, National Institutes of Health, Rockville, MD 20852 USA; 6 Centre for Tropical Medicine & Global Health, Nuffield Department of Medicine, University of Oxford, Oxford, UK

**Keywords:** Malaria, *Plasmodium falciparum*, Anti-malarial resistance, Chloroquine, Amodiaquine, Mefloquine, Artemisinin, Combination therapy, pfcrt, pfmdr1

## Abstract

**Background:**

Declining anti-malarial efficacy of artemisinin-based combination therapy, and reduced *Plasmodium falciparum* susceptibility to individual anti-malarials are being documented across an expanding area of Southeast Asia (SEA). Genotypic markers complement phenotypic studies in assessing the efficacy of individual anti-malarials.

**Methods:**

The markers *pfmdr1* and *pfcrt* were genotyped in parasite samples obtained in 2011–2014 at 14 TRAC (Tracking Resistance to Artemisinin Collaboration) sites in mainland Southeast Asia using a combination of PCR and next-generation sequencing methods.

**Results:**

*Pfmdr1* amplification, a marker of mefloquine and lumefantrine resistance, was highly prevalent at Mae Sot on the Thailand–Myanmar border (59.8% of isolates) and common (more than 10%) at sites in central Myanmar, eastern Thailand and western Cambodia; however, its prevalence was lower than previously documented in Pailin, western Cambodia. The *pfmdr1* Y184F mutation was common, particularly in and around Cambodia, and the F1226Y mutation was found in about half of samples in Mae Sot. The functional significance of these two mutations remains unclear. Other previously documented *pfmdr1* mutations were absent or very rare in the region. The *pfcrt* mutation K76T associated with chloroquine resistance was found in 98.2% of isolates. The CVIET haplotype made up 95% or more of isolates in western SEA while the CVIDT haplotype was common (30–40% of isolates) in north and northeastern Cambodia, southern Laos, and southern Vietnam.

**Conclusions:**

These findings generate cause for concern regarding the mid-term efficacy of artemether–lumefantrine in Myanmar, while the absence of resistance-conferring *pfmdr1* mutations and SVMNT *pfcrt* haplotypes suggests that amodiaquine could be an efficacious component of anti-malarial regimens in SEA.

**Electronic supplementary material:**

The online version of this article (doi:10.1186/s12936-016-1598-6) contains supplementary material, which is available to authorized users.

## Background

Anti-malarial resistance in *Plasmodium falciparum* has originated and spread from Southeast Asia (SEA) on multiple occasions, and the high global prevalence of chloroquine and antifolate resistance has made these drugs ineffective in the vast majority of malaria-endemic areas. In SEA, artemisinin-based combination therapies (ACTs) that combine mefloquine, lumefantrine, or piperaquine with an artemisinin derivative are currently used as front-line treatments. However, emerging resistance to artemisinins [1–5] and their partner drugs is causing ACT cure rates to fall below acceptable levels at an increasing number of sites in SEA [6–10]. When choosing the ideal ACT for a given location, validated molecular resistance markers can provide useful data that complement the results of clinical trials and in vitro studies, and potentially identify resistance trends at a relatively early stage [11].

The global spread of chloroquine resistance was primarily caused by mutant haplotypes in *pfcrt*, often accompanied by additional mutations in *pfmdr1* (corresponding to codons 86, 184, 1034, 1042, and 1246). Paradoxically, some of these *pfmdr1* mutations are associated with hypersensitivity to mefloquine and lumefantrine [12, 13]; accordingly, switching to a drug regimen containing these latter drugs first leads to reselection of *pfmdr1* wild-type alleles [14]. True resistance to mefloquine and lumefantrine then arises through amplification of *pfmdr1* [15, 16], and is associated with clinical failure of the ACT artesunate–mefloquine [15, 17, 18] and artemether–lumefantrine [14].

**Fig. 1:**
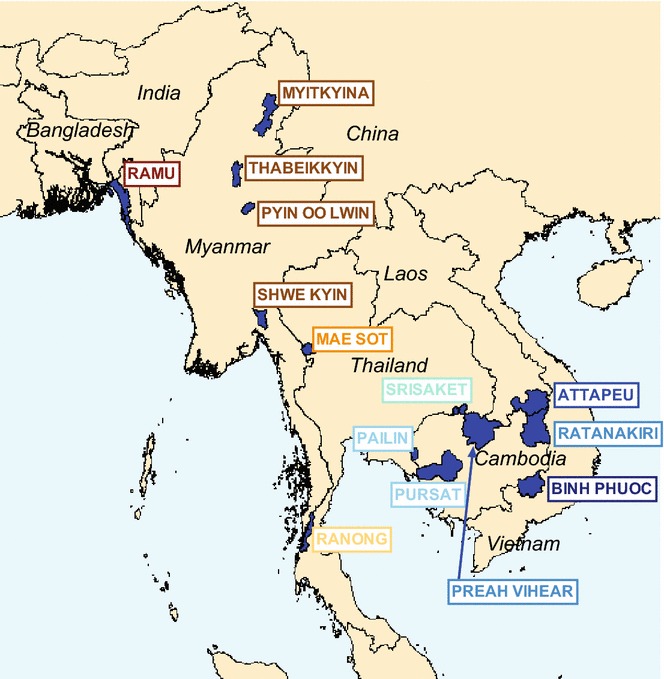
Locations of study sites

The prevalence of different molecular markers of resistance has been described in certain parts of SEA, but not in many other areas, notably Myanmar, the country with the highest malaria caseload. Here, relevant mutations in *pfcrt* and *pfmdr1* were examined in samples from the Tracking Resistance to Artemisinin Collaboration (TRAC) study [5], covering 14 sites in six countries (Fig. 1).

## Methods

### Sample collection

Samples were collected as part of the TRAC study, a clinical trial focused on artemisinin resistance, between May 2011 and April 2013 [5]. Samples from three additional (TRAC Continuation) sites in Myanmar were collected in 2013–2014 (Fig. 1; Table 1).

**Table 1:** Summary of Southeast Asian samples studied

Year	Site	Province	Country	Total samples available	Samples analysed by PCR (Thailand)	Samples analysed by Illumina (UK)	Samples analysed by PCR (USA)^a^
TRAC study							
2012	Ramu	Cox’s Bazar	Bangladesh	51	30	48	
2011–2012	Pailin	Pailin	Cambodia	90	33	84	
2011–2012	Pursat	Pursat	Cambodia	120		98	120
2011–2012	Preah Vihear	Preah Vihear	Cambodia	120		96	120
2011–2012	Ratanakiri	Ratanakiri	Cambodia	120		93	120
2011–2012	Attapeu	Attapeu	Laos	86	37	85	
2012	Shwe Kyin	Bago	Myanmar	64	30	61	
2011–2012	Mae Sot	Tak	Thailand	107	38	104	
2011–2013	Srisaket	Srisaket	Thailand	36	33	21	
2011–2012	Kraburi	Ranong	Thailand	23	23	20	
2011–2012	Bu Gia map	Binh Phuoc	Vietnam	102	29	97	
TRAC continuation study							
2012–2014	Pyin Oo Lwin	Mandalay	Myanmar	29	29		
2013–2014	Thabeikkyin	Mandalay	Myanmar	30	30		
2013	Myitkyina	Kachin	Myanmar	20	20		
			Total	998	332	807	360

^a^Previously described [20]

Samples were analysed by PCR in the Molecular Malaria Laboratory, Faculty of Tropical Medicine, Bangkok, Thailand and by whole-genome sequencing (WGS) at the Wellcome Trust Sanger Institute, Hinxton, UK [19]. PCR was used to assess *pfmdr1* copy number and five well-described single-nucleotide polymorphisms (SNPs) in *pfmdr1* for approximately 30 samples from each site. WGS data covering all SNPs in *pfmdr1* and the classical *pfcrt* haplotypes encoding amino acids 72–76, were available for all samples tested. Because of timing and logistical issues, samples from the three additional sites in Myanmar were only analysed by PCR. *Pfmdr1* copy number measurements for all 120 samples from each of three Cambodian sites (Pursat, Preah Vihear, Ratanakiri) have already been reported [20] and were included in the overall analysis (Table 1).

### DNA processing and whole-genome sequencing

Admission blood samples were anticoagulated in EDTA, washed in PBS, and filtered through a cellulose CF11 column to deplete host leukocytes [21]. Genomic DNA was extracted using QIAamp^®^ DNA Mini Kit (QIAGEN, Germany), following the manufacturer’s instructions. Eluted genomic DNA samples were quantified by PicoGreen analysis and quantitative real-time PCR using the Applied Biosystems StepOne RT-PCR system and frozen at −80 °C.

Samples with more than 50 ng DNA and less than 80% human DNA contamination were submitted for WGS using the Illumina Genome Analyzer II platform. The procedure for sequencing, assembly of sequencing reads, variant calling, quality filtering, and genotype calling has been fully described elsewhere [19, 22]. Each sample was genotyped at each of 926,988 high-quality exonic positions [19], not all of which were polymorphic within the sample set. Naturally this included the *pfcrt* and *pfmdr1* resistance markers. Genome-wide genotype data were used to compute a genetic distance matrix from which a neighbour-joining tree was constructed, as previously described [19].

### Analysis of WGS read depth

In each sample, the *coverage depth* (*c*_*i*_) at each position (i.e., the number of sequencing reads that cover that position in the sample alignment) was computed, from which the *median* coverage of the sample (*c*_*m*_) was determined. The *relative coverage* (*c*_*r*_) at each position was then obtained by *c*_*r*_ = *c*_*i/*_ *c*_*m*_. Since such a large proportion of the *P. falciparum* genome is very unevenly covered [22], *c*_*r*_ was not directly used to estimate copy number. Rather, a number of *reference positions* were identified that exhibited consistent coverage across the complete sample set and were located in *pfmdr1* or genes with similar characteristics.

Seven reference positions were identified in two regions of *pfmdr1* that exhibited even coverage and GC content; their spacing (~250 bp) is such that coverage is not affected by the same reads, and they have very low minor allele frequency (MAF) to minimize the probability of mis-mappings. For each sample, the relative coverage of *pfmdr1* (*c*_*mdr1*_) was estimated as the median of *c*_*r*_ at the *pfmdr1* reference positions.

A further 56 reference positions were identified in genes similar to *pfmdr1*, i.e., with conservation score estimated as previously described [23] in the range 3.2–4.0 (*pfmdr1* conservation score = 3.6), and a single exon of size >3 kbp. The positions were chosen within exonic regions >300 nucleotides devoid of high-frequency SNPs, with similar GC content (~24%) and median coverage to *pfmdr1.* Each reference position was chosen to have a limited coverage range variation across all samples in the MalariaGEN *P. falciparum* Community Project [24], with inter-quartile range (IQR) boundaries within 15% of *c*_*mdr1*_. The selected reference positions are listed in Additional file 1. Coverage statistics at these positions can be visualized using the *P. falciparum* Community Project web application [24]; see Additional file 2.

For each sample, the reference relative coverage (*c*_*ref*_) was calculated as the median of *c*_*r*_ at the 56 reference positions outside *pfmdr1*. The *pfmdr1* copy number was thus estimated to be *N*_*est*_ = *c*_*mdr1/*_ *c*_*ref*_. A plot of the distribution of *N*_*est*_ in the present sample set (Additional file 3) shows clear peaks at integer values. *Pfmdr1* amplification was defined as copy number >1.5.

### *Pfcrt* haplotype determination

Due to the high number of small exons, the low complexity of the introns, and the extreme levels of polymorphism around the key drug-resistance variation site, *pfcrt* is a very difficult gene to assemble from Illumina short-read sequence data, even in otherwise well-covered samples. As a result, the previously used routine genotype calling method [22] is unable to determine *pfcrt* genotypes in many samples. To overcome this problem, a novel bespoke procedure was used for genotyping the *core pfcrt haplotype* (defined as amino acid positions 72–76).

Each flank of the *core pfcrt haplotype* contains a significant number of positions which are invariant in all the *P. falciparum* Community Project samples, forming two invariant *flanking sequences*: TATTATTTATTTAAGTGTA upstream of the *core pfcrt haplotype* and ATTTTTGCTAAAAGAAC downstream. The application samtools V1.2 [25] was used to extract all sequencing reads containing the two flanking sequences (or their reverse complement) from each sample’s alignment. The reads were then aligned against the V3 3D7 *pfcrt* reference sequence (GeneDB PF3D7_0709000), after discarding low-quality reads (i.e., those carrying *phred* scores lower than 20 in the core haplotype codons). The core haplotype for each sample was finally read directly from the resulting alignment.

### PCR methods

*Pfmdr1* amplification undertaken in Thailand utilized Taqman real-time PCR (Rotor Gene 3000; Corbett Research, Australia) following established procedures and using published primers [15, 26]. In each set of reactions, the 3D7 *P. falciparum* strain (single copy) was used as a calibrator (in triplicate). All reaction sets also included a previously derived positive control DNA extract with an estimated *pfmdr1* copy number of 2.3 [27]; any run in which this gave a result of fewer than two or more than three copies was re-tested; in 27 separate runs the mean copy number was 2.34 (95% CI 2.25–2.43, range 2.06–2.83). A negative control (reagents only) was also tested each time. The threshold cycle (Ct) of samples was calculated by the ΔΔCt calculation for the relative quantification of target: ΔΔCt = (Ct *pfmdr1* − Ct *pf β*-*tubulin*) of sample − (Ct *pfmdr1* − Ct *pf β*-*tubulin*) of *P. falciparum* 3D7. Copy number was calculated by the formula = 2^ΔΔCt^. A cut-off copy number of 1.5 was used to define *pfmdr1* amplification. Reactions were repeated whenever the profile did not conform to exponential kinetics, or ΔΔCt spread was >1.5, or the Ct value was >35. To confirm amplification and resolve indeterminate results, samples passing these criteria but with an estimated copy number >1.3 were also re-tested once, the second result counting as final. For the three Cambodian sites where data have already been published, a conservative copy number cut-off of 1.7 was used to define amplification [20].

*Pfmdr1* polymorphism was examined at codons 86, 184, 1034, 1042, and 1246 via PCR-restriction fragment length polymorphism (PCR–RFLP) using an established protocol [28].

Comparison of polymorphism results for samples successfully assessed by both PCR and WGS, based on whether *pfmdr1* was categorized as amplified or not, were analysed by the kappa statistic. To calculate the overall proportion of samples with amplified *pfmdr1* at each site, the WGS-derived result was used where available; otherwise the corresponding PCR result was used (191 samples).

### Ethics

The samples were tested under existing ethical approvals from the TRAC coordinating centre and individual sites [5]; additional ethical approval was obtained from the Ethics Committee of the Faculty of Tropical Medicine, Mahidol University, Bangkok, for laboratory work in Thailand. The TRAC study is registered with ClinicalTrials.gov (NCT01240603).

## Results

### *Pfmdr1* amplification

**Fig. 2:**
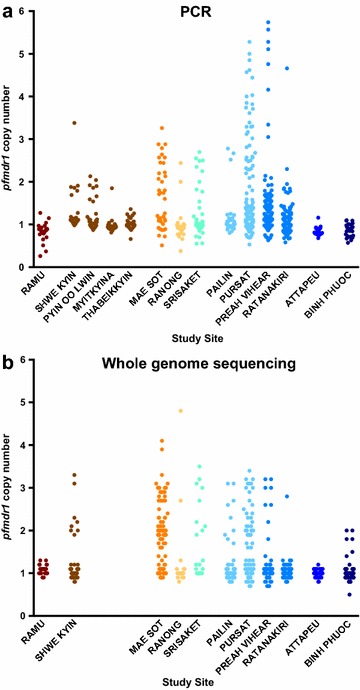
Distribution of *pfmdr1* copy number across sites measured by PCR (**a**) or Illumina sequencing (**b**). The numbers of samples assessed at each site were not the same for the two methods (see Table 1)

**Fig. 3:**
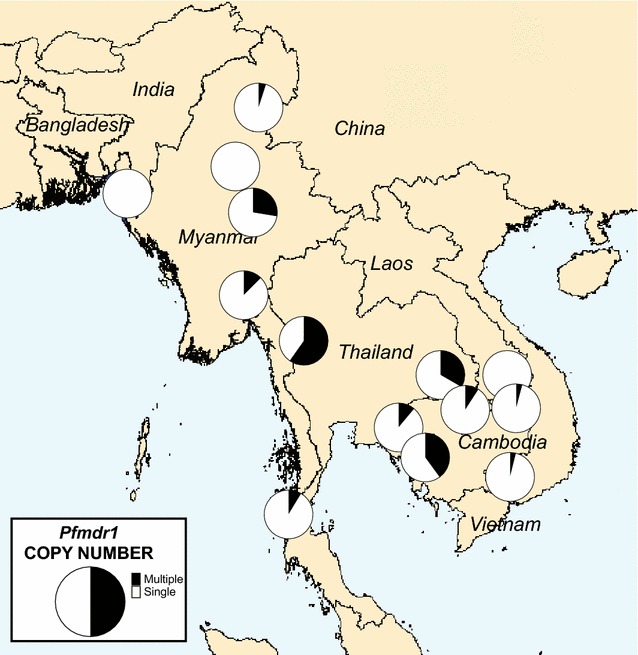
Proportion of isolates with *pfmdr1* amplification at each site

Overall, 998 samples had *pfmdr1* copy number measured by PCR, WGS, or both. PCR-based assessment of *pfmdr1* copy number was successful in 332 samples tested in Thailand, adding to the 360 published results for three Cambodian sites (Table 1; Fig. 2a). *Pfmdr1* copy number was also assessed in 807 samples for which WGS data were available (Fig. 2b). Comparison of results for the samples successfully assessed by both PCR and WGS, based on whether *pfmdr1* was categorized as amplified or not, indicated 93.2% (467/501) agreement between methods (κ = 0.766, 95% CI 0.691–0.841). To calculate the overall proportion of samples with amplified *pfmdr1* at each site, the WGS-derived result was used where available; otherwise the corresponding PCR result was used (191 samples). The proportions of samples with amplification according to site are shown graphically in Fig. 3 and in tabular format in Additional file 4.

Samples with amplified *pfmdr1* were found at all three sites in Thailand, being more prevalent in Mae Sot near the Myanmar border (59.8%) and Srisaket (33.3%) near the Cambodia border (Fig. 3). Amplification was also seen at lower levels in central Myanmar at Shwe Kyin (12.5%) and Pyin Oo Lwin (28.0%). In Cambodia, amplification was common in Pursat (40%) but not at three other sites. The proportion of isolates with amplification was low or zero in Bangladesh, northern Myanmar, Laos, and Vietnam.

**Fig. 4:**
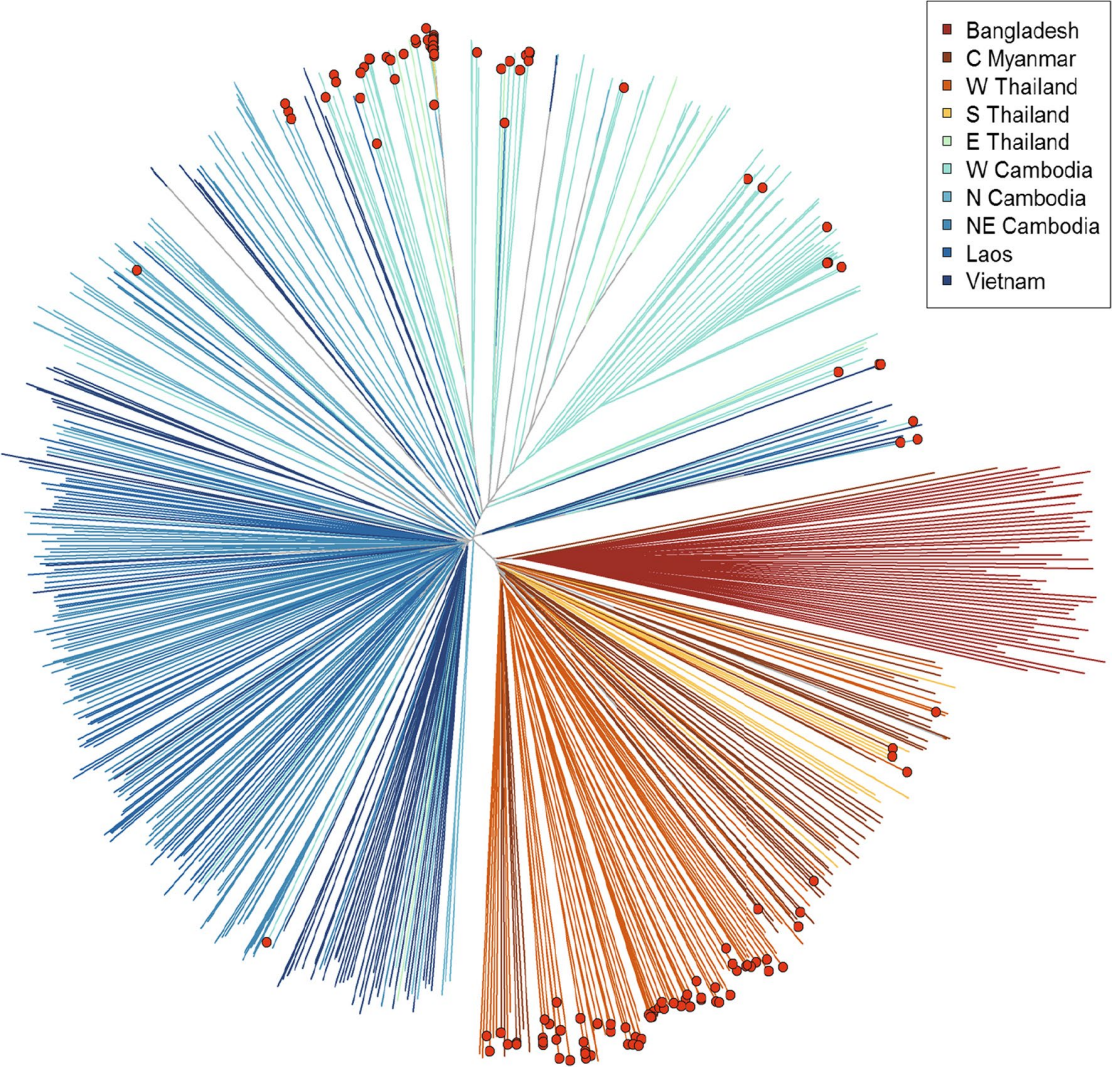
Neighbour-joining tree showing population structure across 11 sites studied by Illumina sequencing. *Branches* with *coloured tip symbols* indicate that the samples have *pfmdr1* amplification

To investigate how the geographical distribution of *pfmdr1* amplification relates to the genetic structure of the parasite population, samples with WGS data were plotted on a neighbour-joining tree, which groups samples according to genome-wide genetic similarity as previously described [19] (Fig. 4). In the ‘western SEA’ compartment [19] (Mae Sot and Ranong at the Thailand–Myanmar border and Shwe Kyin in central Myanmar), *pfmdr1* amplification was evenly dispersed, while in the more structured populations of western and northern Cambodia (Pailin, Pursat, and Preah Vihear) and eastern Thailand (Srisaket), *pfmdr1* amplification tended to cluster within particular branches.

### *Pfmdr1* polymorphism

The polymorphic positions N86Y, Y184F, S1034C, N1042D, and D1246Y were assessed by both PCR–RFLP and Illumina methods. Overall, agreement was 97.4% (κ = 0.825, 95% CI 0.765–0.885), with most disagreements consisting of heterozygous calls by WGS that were assigned a homozygous genotype by PCR–RFLP. Samples that yielded a heterozygous genotype by either method, or for which the two methods produced discordant homozygous genotypes (six calls), were considered as having mixed alleles at that position, and apportioned equally between the two alleles for the purposes of calculating allele frequency. Summary results for all five positions are reported in tabular format in Additional file 5.

**Fig. 5:**
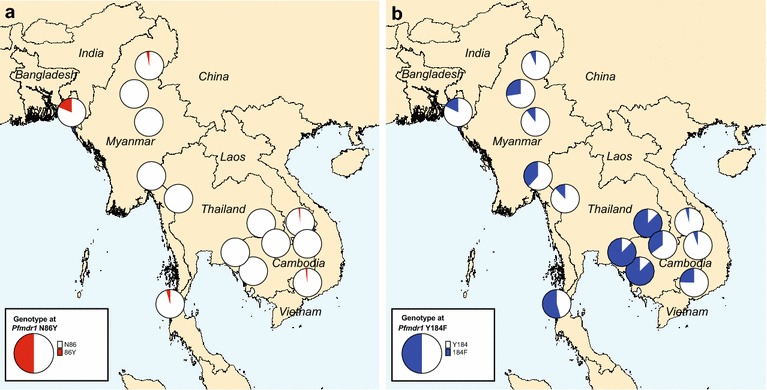
Mutant allele frequency per site for the *pfmdr1* SNPs N86Y (**a**) and Y184F (**b**)

The N86Y mutation was found in 19% of samples in Bangladesh and in fewer than 5% of samples at all other sites (Fig. 5a). The N86F mutation [29] was also observed in three mixed samples in Bangladesh. The Y184F mutation was found in more than 10% of parasites at most sites, being particularly common (85–90% of samples) in western Cambodia (Pailin and Pursat) and eastern Thailand (Srisaket) (Fig. 5b). The N1042D mutation was rare (ten samples including four mixed alleles), while the S1034C and D1246Y mutations were entirely absent from the sample set.

### Other *pfmdr1* polymorphisms

Analysis of WGS data also identified 11 additional *pfmdr1* SNPs distinct from the five SNPs commonly assessed (Additional file 6). While these were generally found at low frequencies, in six cases the derived allele had a frequency of more than 10% in at least one site (Additional file 7). The F1226Y mutation was found in 54% of samples in Mae Sot. The A784L mutation was found in >20% of samples in Pailin, Cambodia and Shwe Kyin, Myanmar.

### *Pfcrt* polymorphism

**Fig. 6:**
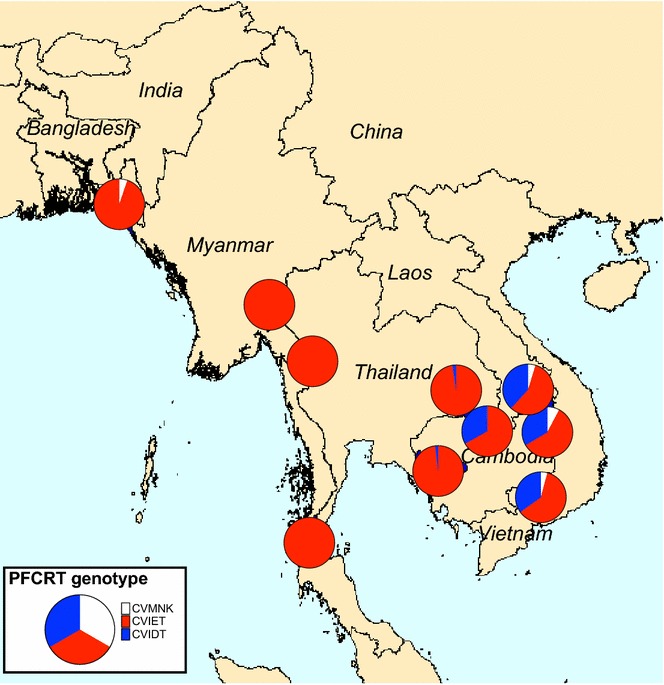
Proportion of isolates with each of three *pfcrt* haplotypes encoding amino acids 72–76 (11 sites). The *pfcrt* SVMNT haplotype was not found in any sample

The great majority (98.2%) of samples across the study contained parasites carrying the key chloroquine resistance mutation K76T; only 14 samples carrying the wild-type allele were distributed across Bangladesh, Laos, northeastern Cambodia, and Vietnam (Fig. 6). At residues 72–76, the CVIET haplotype was predominant (95% or more) in Bangladesh, Myanmar, and Thailand, while the CVIDT haplotype was also common in northern and northeastern Cambodia, Laos, and Vietnam (33–38%). No sample carried the SVMNT haplotype associated with high levels of amodiaquine resistance [30].

## Discussion

This large survey of the molecular markers *pfmdr1* and *pfcrt* offers insights into patterns of anti-malarial partner drug susceptibility across mainland SEA, which reflect the history of anti-malarial use and may guide future therapeutic studies at each location.

*Pfmdr1* amplification is associated with reduced efficacy of both artemether–lumefantrine, globally the most widely used ACT [14], and artesunate–mefloquine. *Pfmdr1* copy number can be assessed by a range of methods [31]. Most surveys of field isolates have used quantitative PCR focused on the individual gene [32], while genome-wide studies of specific laboratory isolates have used microarray-based or next-generation approaches [33–35]. Here, a comparison of PCR-based and next-generation sequencing data in approximately 500 samples showed good agreement between the two approaches.

Artemether–lumefantrine is the currently recommended first-line treatment in Bangladesh, Myanmar, and Laos. The presence of substantial numbers of *P. falciparum* isolates with *pfmdr1* amplification in Shwe Kyin (Bago Province) and Pyin Oo Lwin (Mandalay Province), combined with results from a smaller number of samples obtained from nearby sites [36] indicates that isolates with amplified *pfmdr1* are common in central Myanmar. High levels of *pfmdr1* amplification are also present along the Thailand–Myanmar border, where they have persisted for over two decades [15, 37], although the efficacy of artesunate–mefloquine has dropped to unacceptable levels only within the last 5 years, following the emergence of artemisinin resistance [4, 7, 38].

Isolates with amplified *pfmdr1* were also seen in Ranong (southern Thailand–Myanmar border), consistent with previous data [39–41]. Analysis of genome-wide genetic similarity shows that parasites from the Thailand–Myanmar border and central Myanmar (Shwe Kyin) are related; it is worth noting that most patients enrolled in Mae Sot travelled from Myanmar, with the catchment area extending as far as 40 km inside the border [7]. Samples with amplified *pfmdr1* are evenly distributed within this parasite population compartment, rather than associated with any specific sub-population. At the two sites in northern Myanmar (Thabeikkyin and Myitkyina), *pfmdr1* amplification appears to be at low prevalence, consistent with previous data from nearby border areas [42]. However, there is clearly a need for close observation of the efficacy of artemether–lumefantrine in Myanmar given that reduced parasite clearance rates after ACT have been observed in border areas [43, 44] and K13-propeller mutations that confer artemisinin resistance are widespread [45].

*Pfmdr1* amplification was also present at Srisaket in eastern Thailand near the Cambodian border, consistent with continued mefloquine usage, following its adoption as first-line therapy in 2007. Given the widespread nature of samples with K13-propeller mutations and slow clearance following artesunate treatment [5], there is concern that artesunate–mefloquine efficacy will be compromised across a wider area of Thailand in the near future. These data match previously published data from a distinct area of the Thailand–Cambodia border [39, 46].

*Pfmdr1* amplification was found at Pailin and Pursat in western Cambodia, although for Pailin this was at lower levels compared to 2004 [17] when artesunate–mefloquine had unacceptably low efficacy [6]. This likely reflects the 2008–2010 change of policy, leading to the adoption of dihydroartemisinin–piperaquine as the frontline ACT across Cambodia. Since the TRAC study, there has been further reduction in the prevalence of parasites with increased *pfmdr1* copy number in western and northern Cambodian [10, 47, 48]. In Cambodia, *pfmdr1* amplification tended to be found within specific sub-populations in these locations, probably as a result of pronounced population structure caused by the expansion of artemisinin-resistant founder populations [49].

Laos has been using artemether–lumefantrine since 2001, but there was no evidence of *pfmdr1* amplification. This is likely to reflect the introduction of artemether–lumefantrine as national policy without a prior period of mefloquine monotherapy, with maintained artemisinin sensitivity up to the time of this study [5], and is consistent with the documented high efficacy of both artemether–lumefantrine and artesunate–mefloquine [50]. Vietnam has a distinct history of antimalarial use, with dihydroartemisinin–piperaquine deployed as first-line therapy in 2005 (taking over from artesunate plus mefloquine). Consistent with the longstanding absence of mefloquine or lumefantrine from antimalarial therapy, few parasites from Vietnam showed *pfmdr1* amplification.

SNPs in *pfmdr1* may provide additional relevant information for guiding anti-malarial policy and planning further studies. The N86Y polymorphism is of relevance to aminoquinoline sensitivity (see below). Y184F polymorphism was most common, especially in western Cambodia and eastern Thailand, and WGS data also revealed other *pfmdr1* mutations (e.g., the F1226Y mutation prevalent in Mae Sot). Both of these mutations have been associated with decreased in vitro susceptibility to mefloquine [51, 52], although most studies have not found Y184F to be significantly associated with changes in drug responses [53, 54]. Further studies are needed to determine whether these mutations are being naturally selected for drug resistance or other phenotypes.

The *pfcrt* K76T mutation was at or near fixation in all study sites, with the CVIET haplotype dominant in western SEA and the CVIDT haplotype found in approximately one-third of isolates from northern and northeastern Cambodia [55], southern Laos, and southern Vietnam. The SVMNT *pfcrt* haplotype associated with high-grade amodiaquine resistance was not found in any location, in contrast to a study in 2003 in northern Laos [56] and other areas of SEA [57]. The *pfmdr1* N86Y mutation, which plays a role in amodiaquine resistance [13, 30], has all but disappeared from the region following abandonment of chloroquine and the use of mefloquine in the period leading up to this study, and in line with other observations [58]. For these reasons, amodiaquine may show some useful efficacy in SEA. This is also consistent with the acceptable clinical efficacy of artesunate–amodiaquine in some [59, 60], but not all [61], parts of SEA within the last decade. Furthermore, the inverse correlation between susceptibility to 4-aminoquinolines and mefloquine [62, 63] offers the potential to combine two partners with opposing resistance mechanisms within a novel ‘triple’ ACT, artemether–lumefantrine plus amodiaquine [64]; this is currently under investigation in the follow-up study to TRAC (ClinicalTrials.gov NCT02453308).

## Conclusions

In summary, these data offer new insights into partner drug resistance patterns across a large area of SEA. The finding that *pfmdr1* amplification, associated with mefloquine and lumefantrine resistance, extends into central Myanmar is concerning and highlights the need for close observation of the efficacy of artemether–lumefantrine in Myanmar. In contrast, the reduction in *pfmdr1* amplification in western Cambodia and lower areas of the Greater Mekong Subregion supports the use (or re-use) of artesunate–mefloquine in areas where dihydroartemisinin–piperaquine efficacy is unacceptable. Finally, SNP patterns in *pfmdr1* and *pfcrt* suggest that amodiaquine may improve treatment efficacy if it can be practically incorporated into anti-malarial regimens.

## Additional files

**Additional file 1.** Reference positions used to estimate *pfmdr1* copy number from WGS coverage data.

**Additional file 2.** Screenshot of the MalariaGEN *P. falciparum* Community Project web application (http://www.malariagen.net/apps/pf/4.0/). Location of six reference positions located within gene *PF3D7_1455600* (ferlin, putative).

**Additional file 3.** Histogram of estimated *pfmdr1* copy number in the present dataset, estimated from WGS reads coverage. Each bin represents an estimate value interval of 0.1. A kernel density estimate of these data is overlaid (dark blue curve) to show the location of peaks, derived from the density() function in the R stats package, using a Gaussian kernel with standard deviation 0.15. There are clear peaks in sample numbers at integer values of *pfmdr1* copy number (*N*_*est*_ = {1, 2, 3}), and troughs at intermediate positions, consistent with the expected distribution of *pfmdr1* copy number values.

**Additional file 4.** Proportion of samples with amplified *pfmdr1*, based on a combination of PCR and Illumina-based sequencing.

**Additional file 5.** Numbers of isolates with pure wild-type, mutant or mixed alleles at five well-described polymorphic sites in *pfmdr1* (combination of PCR–RFLP and Illumina methods). *Three samples were mixed N86/86F infections.

**Additional file 6.** Numbers of isolates with pure wild-type, mutant, or mixed alleles at eleven additional polymorphic sites in *pfmdr1* (Illumina method only).

**Additional file 7.** Proportions of mutant *pfmdr1* alleles at six other polymorphic positions (11 study sites).
