# Correction to: Analysis of anti-malarial resistance markers in* pfmdr1* and* pfcrt* across Southeast Asia in the Tracking Resistance to Artemisinin Collaboration

**DOI:** 10.1186/s12936-018-2464-5

**Published:** 2018-09-10

**Authors:** Krongkan Srimuang, Olivo Miotto, Pharath Lim, Rick M. Fairhurst, Dominic P. Kwiatkowski, Charles J. Woodrow, Mallika Imwong, for the Tracking Resistance to Artemisinin Collaboration, Tinh Hien Tran, Nguyen Thanh Thuy, Ye Htut, Khin Lin, Kyaw Myo Tun, Tin M. Hlaing, Mayfong Mayxay, Paul N. Newton, Md. Abul Faiz, Aung Pyae Phyo, François Nosten, Elizabeth A. Ashley, Mehul Dhorda, Sasithon Pukrittayakamee, Frank Smithuis, Nicholas P. J. Day, Arjen M. Dondorp, Nicholas J. White

**Affiliations:** 1 Department of Molecular Tropical Medicine and Genetics, Faculty of Tropical Medicine, Mahidol University, Bangkok, Thailand; 2 Mahidol Oxford Tropical Medicine Research Unit, Bangkok, Thailand; 3 Wellcome Trust Sanger Institute, Hinxton, UK; 4 Medical Research Council (MRC) Centre for Genomics and Global Health, University of Oxford, Oxford, UK; 5 Laboratory of Malaria and Vector Research, National Institute of Allergy and Infectious Diseases, National Institutes of Health, Rockville, MD 20852 USA; 6 Centre for Tropical Medicine & Global Health, Nuffield Department of Medicine, University of Oxford, Oxford, UK

## Correction to: Malar J (2016) 15:541 10.1186/s12936-016-1598-6

Following publication of the original article [1], one of the authors has highlighted an xml-related discrepancy concerning the author group titled ‘Additional Tracking Resistance to Artemisinin Collaboration authors (TRAC Group Authorship)’, listed under the Acknowledgements section.

The effect of this discrepancy is that the author group is not displayed in the version of the article [1] on the indexing service PubMed.

We apologize for any inconvenience caused by this processing error.
