# Supplementary material for: Analysis of anti-malarial resistance markers in pfmdr1 and pfcrt across Southeast Asia in the Tracking Resistance to Artemisinin Collaboration
Source: Malar J. 2016 Nov 8;15:541. doi: 10.1186/s12936-016-1598-6 (PMC5101715; doi:10.1186/s12936-016-1598-6)
Supplement: Supplementary file 1 — Additional file 1. Reference positions used to estimate pfmdr1 copy number from WGS coverage data. [file 12936_2016_1598_MOESM1_ESM.docx]

Additional File 1: Reference positions used to estimate *pfmdr1* copy number from WGS coverage data

| Gene | GeneDescription | Chromosome | Positions |
| --- | --- | --- | --- |
| PF3D7_0523000 | multidrug resistance protein (MDR1) | 5 | 958336 |
|  |  |  | 958741 |
|  |  |  | 960331 |
|  |  |  | 960607 |
|  |  |  | 960962 |
|  |  |  | 961193 |
|  |  |  | 961465 |
| PF3D7_0706000 | conserved Plasmodium protein, unknown function | 7 | 294830 |
|  |  |  | 294995 |
|  |  |  | 295268 |
|  |  |  | 295511 |
|  |  |  | 295772 |
|  |  |  | 296168 |
| PF3D7_0802300 | rRNA processing WD-repeat protein, putative | 8 | 167615 |
|  |  |  | 167912 |
|  |  |  | 169130 |
|  |  |  | 169298 |
| PF3D7_0905300 | Motor dynein heavy chain, putative | 9 | 252284 |
|  |  |  | 252927 |
|  |  |  | 253291 |
|  |  |  | 254848 |
|  |  |  | 255309 |
|  |  |  | 257128 |
|  |  |  | 258241 |
| PF3D7_1023100 | dynein heavy chain, putative | 10 | 965021 |
|  |  |  | 971840 |
|  |  |  | 972263 |
|  |  |  | 972632 |
|  |  |  | 972941 |
| PF3D7_1206600 | DNA-directed RNA polymerase III subunit, putative | 12 | 295836 |
|  |  |  | 296094 |
|  |  |  | 297460 |
| PF3D7_1227800 | histone S-adenosyl methyltransferase, putative | 12 | 1135810 |
|  |  |  | 1135993 |
|  |  |  | 1136278 |
| PF3D7_1360800 | falcilysin (FLN) | 13 | 2436367 |
|  |  |  | 2436642 |
|  |  |  | 2437670 |
|  |  |  | 2437953 |
|  |  |  | 2438155 |
| PF3D7_1364300 | pre-mRNA-splicing factor ATP-dependent RNA helicase PRP16, putative (PRP16) | 13 | 2581214 |
|  |  |  | 2581430 |
|  |  |  | 2581993 |
| PF3D7_1408400 | DNA-repair helicase, putative | 14 | 316129 |
|  |  |  | 316417 |
|  |  |  | 316834 |
| PF3D7_1426700 | phosphoenolpyruvate carboxylase, putative (PEPC) | 14 | 1038015 |
|  |  |  | 1038159 |
| PF3D7_1435300 | NAD(P)H-dependent glutamate synthase, putative | 14 | 1424634 |
|  |  |  | 1428039 |
|  |  |  | 1430043 |
| PF3D7_1455600 | ferlin, putative | 14 | 2272519 |
|  |  |  | 2272885 |
|  |  |  | 2275197 |
|  |  |  | 2275756 |
|  |  |  | 2276227 |
|  |  |  | 2276497 |
| PF3D7_1461900 | valine-tRNA ligase, putative | 14 | 2516996 |
|  |  |  | 2517396 |
|  |  |  | 2517690 |
| PF3D7_1475500 | LCCL domain-containing protein (CCp1) | 14 | 3110587 |
|  |  |  | 3111013 |
|  |  |  | 3111451 |
