# Supplementary material for: Analysis of anti-malarial resistance markers in pfmdr1 and pfcrt across Southeast Asia in the Tracking Resistance to Artemisinin Collaboration
Source: Malar J. 2016 Nov 8;15:541. doi: 10.1186/s12936-016-1598-6 (PMC5101715; doi:10.1186/s12936-016-1598-6)
Supplement: Supplementary file 2 — Additional file 2. Screenshot of the MalariaGEN P. falciparum Community Project web application (http://www.malariagen.net/apps/pf/4.0/). Location of six reference positions located within gene PF3D7_1455600 (ferlin, putative). [file 12936_2016_1598_MOESM2_ESM.docx]

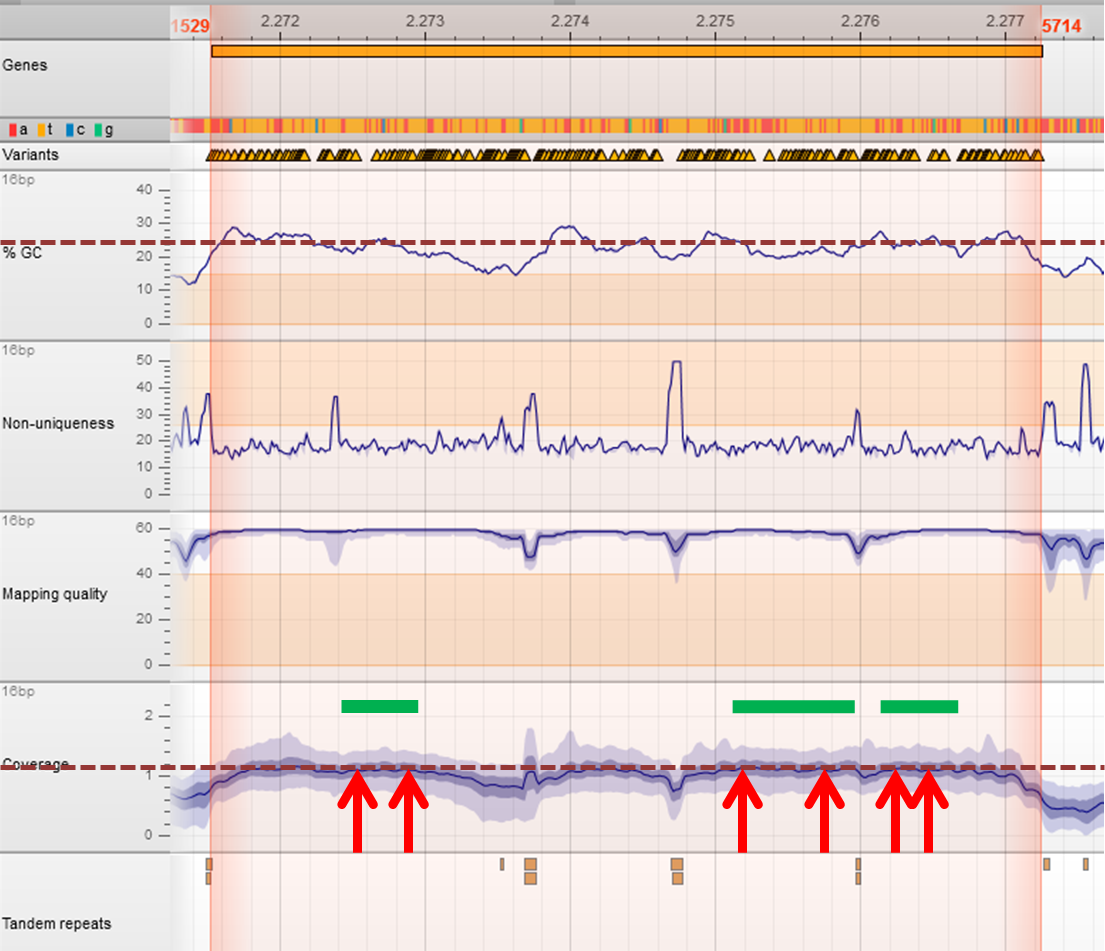


Additional File 2: Screenshot of the MalariaGEN P. falciparum Community Project web application (<http://www.malariagen.net/apps/pf/4.0/>), showing the location of six reference positions located within gene PF3D7_1455600 (ferlin, putative). The display tracks show, from top to bottom: the position within chromosome 14; the extent of the gene exon; the location of all SNPs genotyped in our dataset within the gene; the GC content of a typical mapped read; the non-uniqueness score, a measure of the likelihood that the local sequence is also found elsewhere in the *P. falciparum* genome (24); the read mapping quality; the coverage variation across the sample set, with shading showing the inter-quartile range (dark blue) and the 5-95% range (light blue); and the location of low-complexity tandem repeats. Three green bands indicate regions of the gene where the conditions for selecting reference positions are met (note the narrow coverage variation), and the six arrows show the location of the six reference positions in this gene.
