# Supplementary figures and images for: Analysis of anti-malarial resistance markers in pfmdr1 and pfcrt across Southeast Asia in the Tracking Resistance to Artemisinin Collaboration
Source: Malar J. 2016 Nov 8;15:541. doi: 10.1186/s12936-016-1598-6 (PMC5101715; doi:10.1186/s12936-016-1598-6)

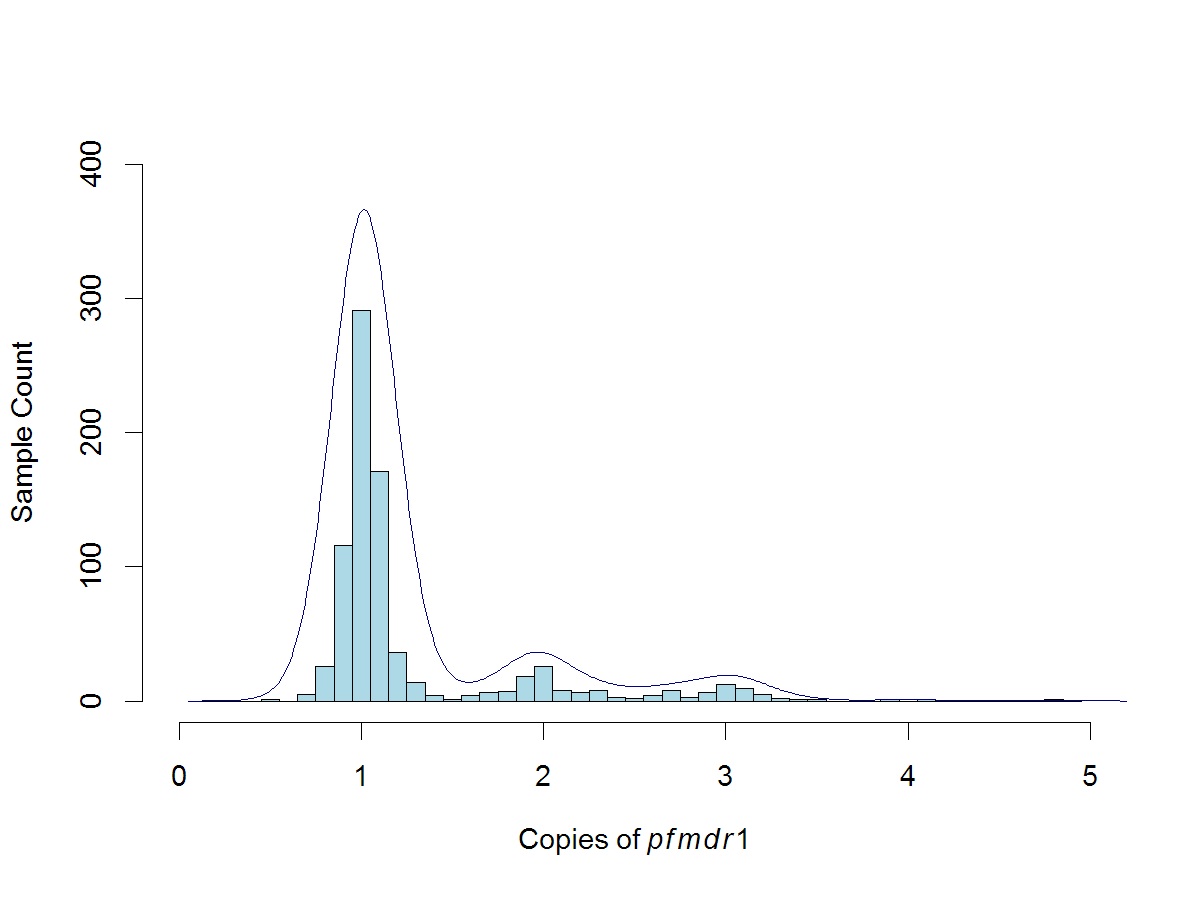

Supplement: Supplementary file 3 — Additional file 3. Histogram of estimated pfmdr1 copy number in the present dataset, estimated from WGS reads coverage. Each bin represents an estimate value interval of 0.1. A kernel density estimate of these data is overlaid (dark blue curve) to show the location of peaks, derived from the density() function in the R stats package, using a Gaussian kernel with standard deviation 0.15. There are clear peaks in sample numbers at integer values of pfmdr1 copy number (N est = {1, 2, 3}), and troughs at intermediate positions, consistent with the expected distribution of pfmdr1 copy number values. [file 12936_2016_1598_MOESM3_ESM.jpg]

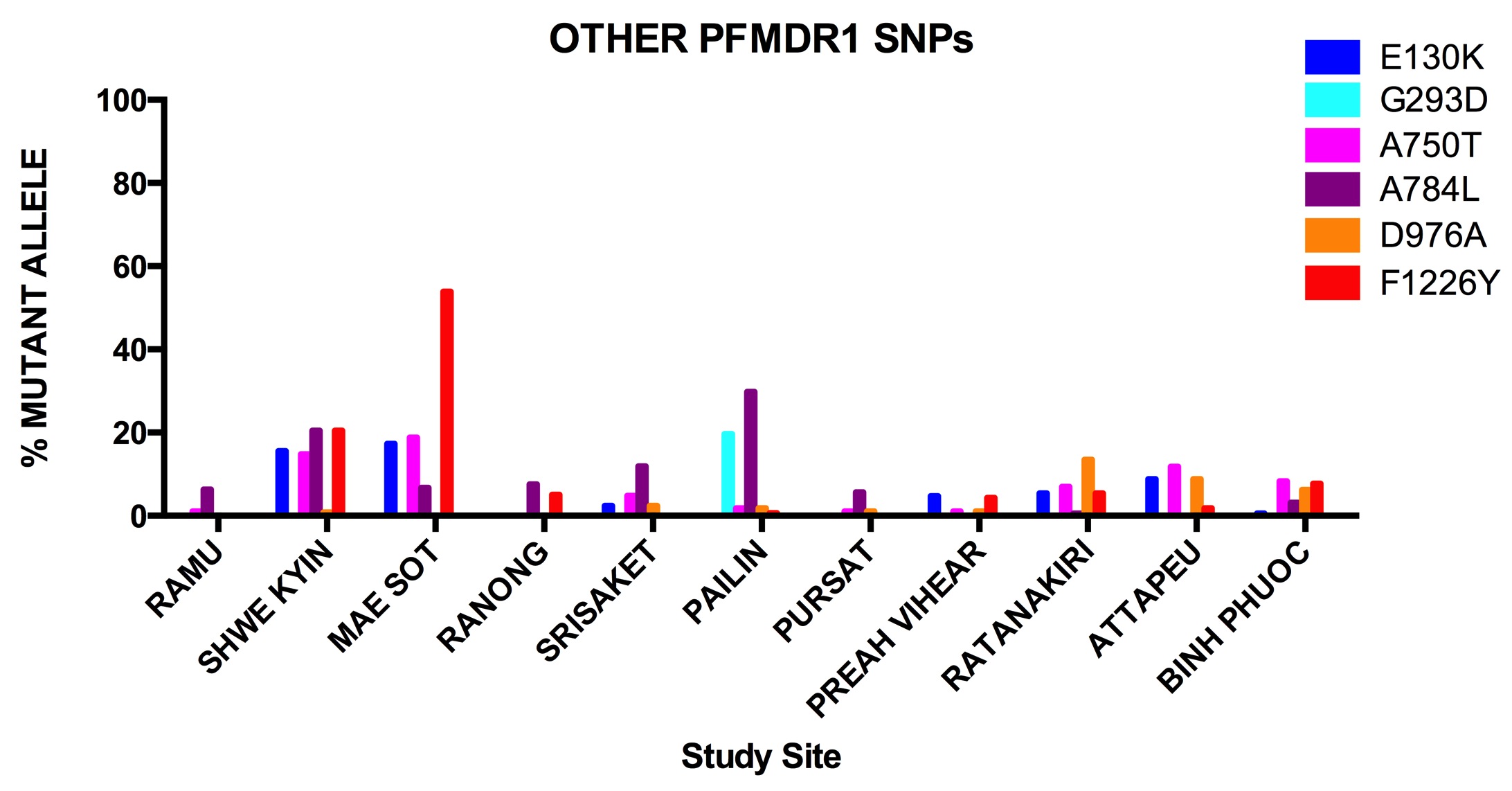

Supplement: Supplementary file 7 — Additional file 7. Proportions of mutant pfmdr1 alleles at six other polymorphic positions (11 study sites). [file 12936_2016_1598_MOESM7_ESM.jpg]
