# Supplementary material for: Analysis of anti-malarial resistance markers in pfmdr1 and pfcrt across Southeast Asia in the Tracking Resistance to Artemisinin Collaboration
Source: Malar J. 2016 Nov 8;15:541. doi: 10.1186/s12936-016-1598-6 (PMC5101715; doi:10.1186/s12936-016-1598-6)
Supplement: Supplementary file 4 — Additional file 4. Proportion of samples with amplified pfmdr1, based on a combination of PCR and Illumina-based sequencing. [file 12936_2016_1598_MOESM4_ESM.docx]

Additional File 4: Proportion of samples with amplified *pfmdr1*, based on a combination of PCR and Illumina-based sequencing.

| **Site** | **Province** | **Country** | **Single** | **Amplified** | **%** |
| --- | --- | --- | --- | --- | --- |
| Ramu | Cox's Bazar | Bangladesh | 51 | 0 | 0.0% |
| Pailin | Pailin | Cambodia | 80 | 10 | 11.1% |
| Pursat | Pursat | Cambodia | 72 | 48 | 40.0% |
| Preah Vihear | Preah Vihear | Cambodia | 109 | 11 | 9.2% |
| Ratanakiri | Ratanakiri | Cambodia | 115 | 5 | 4.2% |
| Attapeu | Attapeu | Laos | 86 | 0 | 0.0% |
| Shwe Kyin | Bago | Myanmar | 56 | 8 | 12.5% |
| Mae Sot | Tak | Thailand | 43 | 64 | 59.8% |
| Srisaket | Srisaket | Thailand | 24 | 12 | 33.3% |
| Kraburi | Ranong | Thailand | 21 | 2 | 8.7% |
| Bu Gia Map | Binh Phuoc | Vietnam | 98 | 4 | 3.9% |
| TRAC+ |  |  |  |  |  |
| Pyin Oo Lwin | Mandalay | Myanmar | 21 | 8 | 27.6% |
| Thabeikkyin | Mandalay | Myanmar | 30 | 0 | 0.0% |
| Myitkyina | Kachin | Myanmar | 19 | 1 | 5.0% |
| Total |  |  | **825** | **173** | 19.7% |
