# Supplementary material for: Analysis of anti-malarial resistance markers in pfmdr1 and pfcrt across Southeast Asia in the Tracking Resistance to Artemisinin Collaboration
Source: Malar J. 2016 Nov 8;15:541. doi: 10.1186/s12936-016-1598-6 (PMC5101715; doi:10.1186/s12936-016-1598-6)
Supplement: Supplementary file 5 — Additional file 5. Numbers of isolates with pure wild-type, mutant or mixed alleles at five well-described polymorphic sites in pfmdr1 (combination of PCR–RFLP and Illumina methods). *Three samples were mixed N86/86F infections. [file 12936_2016_1598_MOESM5_ESM.docx]

Additional File 5: Numbers of isolates with pure wild-type, mutant or mixed alleles at five well-described polymorphic sites in *pfmdr1* (combination of PCR-RFLP and Illumina methods). *Three samples were mixed N86/86F infections.

| **SNP** | **SEQUENCE** | **RAMU** | | **SHWE KYIN** | | **PYIN OO LWIN** | | **THABEIKKYIN** | | **MYITKYINAR** | | **MAE SOT** | | **RANONG** | | **SRISAKET** | | **PAILIN** | | **PURSAT** | | **PREAH VIHEAR** | | **RATANAKIRI** | | **ATTAPEU** | | **BINH PHUOC** | |  |
| --- | --- | --- | --- | --- | --- | --- | --- | --- | --- | --- | --- | --- | --- | --- | --- | --- | --- | --- | --- | --- | --- | --- | --- | --- | --- | --- | --- | --- | --- | --- |
| N86Y | Wild-type | | 39 | | 64 | | 29 | | 20 | | 29 | | 107 | | 22 | | 36 | | 90 | | 98 | | 95 | | 93 | | 83 | | 99 | |
|  | Heterozygote | | 5 | | 0 | | 0 | | 0 | | 1 | | 1 | | 1 | | 0 | | 0 | | 0 | | 0 | | 0 | | 3 | | 2 | |
|  | Mutant | | 7 | | 0 | | 0 | | 0 | | 0 | | 0 | | 0 | | 0 | | 0 | | 0 | | 0 | | 0 | | 0 | | 1 | |
|  | Mutant allele % | | 18.6% | | - | | - | | - | | 1.7% | | 0.5% | | 2.2% | | - | | - | | - | | - | | - | | 1.7% | | 2.0% | |
| Y184F | Wild-type | | 37 | | 36 | | 26 | | 14 | | 28 | | 90 | | 9 | | 4 | | 7 | | 6 | | 56 | | 86 | | 81 | | 71 | |
|  | Heterozygote | | 10 | | 7 | | 0 | | 1 | | 0 | | 8 | | 3 | | 1 | | 8 | | 13 | | 11 | | 4 | | 3 | | 12 | |
|  | Mutant | | 4 | | 21 | | 3 | | 5 | | 2 | | 9 | | 11 | | 31 | | 75 | | 79 | | 28 | | 3 | | 2 | | 19 | |
|  | Mutant allele % | | 17.6% | | 38.3% | | 10.3% | | 27.5% | | 6.7% | | 12.1% | | 54.3% | | 87.5% | | 87.8% | | 87.2% | | 35.3% | | 5.4% | | 4.1% | | 24.5% | |
| S1034C | Wild-type | | 51 | | 64 | | 29 | | 20 | | 30 | | 76 | | 23 | | 36 | | 90 | | 98 | | 96 | | 93 | | 86 | | 102 | |
|  | Heterozygote | | 0 | | 0 | | 0 | | 0 | | 0 | | 0 | | 0 | | 0 | | 0 | | 0 | | 0 | | 0 | | 0 | | 0 | |
|  | Mutant | | 0 | | 0 | | 0 | | 0 | | 0 | | 0 | | 0 | | 0 | | 0 | | 0 | | 0 | | 0 | | 0 | | 0 | |
|  | Mutant allele % | | - | | - | | - | | - | | - | | - | | - | | - | | - | | - | | - | | - | | - | | - | |
| N1042D | Wild-type | | 51 | | 63 | | 29 | | 20 | | 30 | | 106 | | 21 | | 36 | | 90 | | 96 | | 94 | | 91 | | 86 | | 101 | |
|  | Heterozygote | | 0 | | 0 | | 0 | | 0 | | 0 | | 1 | | 2 | | 0 | | 0 | | 0 | | 0 | | 0 | | 0 | | 1 | |
|  | Mutant | | 0 | | 1 | | 0 | | 0 | | 0 | | 0 | | 0 | | 0 | | 0 | | 1 | | 2 | | 2 | | 0 | | 0 | |
|  | Mutant allele % | | - | | 1.6% | | - | | - | | - | | 0.5% | | 4.3% | | - | | - | | 1.0% | | 2.1% | | 2.2% | | - | | 0.5% | |
| D1246Y | Wild-type | | 51 | | 64 | | 29 | | 20 | | 30 | | 76 | | 23 | | 36 | | 90 | | 98 | | 96 | | 93 | | 86 | | 102 | |
|  | Heterozygote | | 0 | | 0 | | 0 | | 0 | | 0 | | 0 | | 0 | | 0 | | 0 | | 0 | | 0 | | 0 | | 0 | | 0 | |
|  | Mutant | | 0 | | 0 | | 0 | | 0 | | 0 | | 0 | | 0 | | 0 | | 0 | | 0 | | 0 | | 0 | | 0 | | 0 | |
|  | Mutant allele % | | - | | - | | - | | - | | - | | - | | - | | - | | - | | - | | - | | - | | - | | - | |
