# Supplementary material for: Analysis of anti-malarial resistance markers in pfmdr1 and pfcrt across Southeast Asia in the Tracking Resistance to Artemisinin Collaboration
Source: Malar J. 2016 Nov 8;15:541. doi: 10.1186/s12936-016-1598-6 (PMC5101715; doi:10.1186/s12936-016-1598-6)
Supplement: Supplementary file 6 — Additional file 6. Numbers of isolates with pure wild-type, mutant, or mixed alleles at eleven additional polymorphic sites in pfmdr1 (Illumina method only). [file 12936_2016_1598_MOESM6_ESM.docx]

Additional File 6: Numbers of isolates with pure wild-type, mutant or mixed alleles at eleven additional polymorphic sites in *pfmdr1* (Illumina method only).

| **SNP** | **SEQUENCE** | **RAMU** | **SHWE KYIN** | **MAE SOT** | **RANONG** | **SRISAKET** | **PAILIN** | **PURSAT** | **PREAH VIHEAR** | **RATANAKIRI** | **ATTAPEU** | **BINH PHUOC** |
| --- | --- | --- | --- | --- | --- | --- | --- | --- | --- | --- | --- | --- |
| E130K | Wild-type | 48 | 50 | 76 | 20 | 20 | 83 | 97 | 90 | 87 | 72 | 96 |
|  | Mixed | 0 | 3 | 20 | 0 | 1 | 0 | 0 | 3 | 2 | 11 | 1 |
|  | Mutant | 0 | 8 | 8 | 0 | 0 | 0 | 0 | 3 | 4 | 2 | 0 |
|  | Mutant allele % | - | 15.6% | 17.3% | - | 2.4% | - | - | 4.7% | 5.4% | 8.8% | 0.5% |
| G293D | Wild-type | 48 | 61 | 104 | 20 | 21 | 67 | 98 | 95 | 93 | 85 | 97 |
|  | Mixed | 0 | 0 | 0 | 0 | 0 | 1 | 0 | 0 | 0 | 0 | 0 |
|  | Mutant | 0 | 0 | 0 | 0 | 0 | 16 | 0 | 0 | 0 | 0 | 0 |
|  | Mutant allele % | - | - | - | - | - | 19.6% | - | - | - | - | - |
| A750T | Wild-type | 47 | 50 | 73 | 20 | 19 | 82 | 97 | 95 | 86 | 71 | 87 |
|  | Mixed | 1 | 4 | 23 | 0 | 2 | 1 | 0 | 0 | 1 | 8 | 4 |
|  | Mutant | 0 | 7 | 8 | 0 | 0 | 1 | 1 | 1 | 6 | 6 | 6 |
|  | Mutant allele % | 1.0% | 14.8% | 18.8% | - | 4.8% | 1.8% | 1.0% | 1.0% | 7.0% | 11.8% | 8.2% |
| N752H | Wild-type | 48 | 61 | 104 | 20 | 21 | 84 | 98 | 96 | 93 | 85 | 91 |
|  | Mixed | 0 | 0 | 0 | 0 | 0 | 0 | 0 | 0 | 0 | 0 | 2 |
|  | Mutant | 0 | 0 | 0 | 0 | 0 | 0 | 0 | 1 | 0 | 0 | 4 |
|  | Mutant allele % | - | - | - | - | - | - | - | 1.0% | - | - | 5.2% |
| A784L | Wild-type | 45 | 47 | 90 | 18 | 17 | 57 | 91 | 96 | 92 | 85 | 94 |
|  | Mixed | 0 | 3 | 14 | 1 | 3 | 4 | 3 | 0 | 1 | 0 | 0 |
|  | Mutant | 3 | 11 | 0 | 1 | 1 | 23 | 4 | 0 | 0 | 0 | 3 |
|  | Mutant allele % | 6.3% | 20.5% | 6.7% | 7.5% | 11.9% | 29.8% | 5.6% | - | 0.5% | - | 3.1% |
| D976A | Wild-type | 48 | 60 | 104 | 20 | 20 | 82 | 97 | 95 | 76 | 74 | 88 |
|  | Heterozygote | 0 | 1 | 0 | 0 | 1 | 1 | 0 | 0 | 9 | 7 | 6 |
|  | Mutant | 0 | 0 | 0 | 0 | 0 | 1 | 1 | 1 | 8 | 4 | 3 |
|  | Mutant allele % | - | 0.8% | - | - | 2.4% | 1.8% | 1.0% | 1.0% | 13.4% | 8.8% | 6.2% |
| F1068L | Wild-type | 48 | 61 | 104 | 20 | 21 | 80 | 97 | 96 | 93 | 85 | 97 |
|  | Heterozygote | 0 | 0 | 0 | 0 | 0 | 1 | 0 | 0 | 0 | 0 | 0 |
|  | Mutant | 0 | 0 | 0 | 0 | 0 | 3 | 0 | 0 | 0 | 0 | 0 |
|  | Mutant allele % | - | - | - | - | - | 4.2% | - | - | - | - | - |
| V1109I | Wild-type | 47 | 61 | 103 | 20 | 21 | 84 | 98 | 89 | 85 | 81 | 95 |
|  | Heterozygote | 1 | 0 | 1 | 0 | 0 | 0 | 0 | 3 | 3 | 2 | 2 |
|  | Mutant | 0 | 0 | 0 | 0 | 0 | 0 | 0 | 3 | 5 | 2 | 0 |
|  | Mutant allele % | 1.0% | - | 0.5% | - | - | - | - | 4.7% | 7.0% | 3.5% | 1.0% |
| F1226Y | Wild-type | 48 | 46 | 33 | 18 | 21 | 83 | 98 | 89 | 87 | 83 | 88 |
|  | Heterozygote | 0 | 5 | 30 | 2 | 0 | 1 | 0 | 2 | 2 | 1 | 3 |
|  | Mutant | 0 | 10 | 41 | 0 | 0 | 0 | 0 | 3 | 4 | 1 | 6 |
|  | Mutant allele % | - | 20.5% | 53.8% | 5.0% | - | 0.6% | - | 4.3% | 5.4% | 1.8% | 7.7% |
| G1314D | Wild-type | 48 | 61 | 104 | 20 | 21 | 81 | 96 | 96 | 93 | 85 | 97 |
|  | Heterozygote | 0 | 0 | 0 | 0 | 0 | 0 | 0 | 0 | 0 | 0 | 0 |
|  | Mutant | 0 | 0 | 0 | 0 | 0 | 3 | 1 | 0 | 0 | 0 | 0 |
|  | Mutant allele % | - | - | - | - | - | 3.6% | 1.0% | - | - | - | - |
| R1328K | Wild-type | 48 | 61 | 104 | 20 | 20 | 84 | 98 | 95 | 93 | 77 | 95 |
|  | Heterozygote | 0 | 0 | 0 | 0 | 0 | 0 | 0 | 0 | 0 | 1 | 2 |
|  | Mutant | 0 | 0 | 0 | 0 | 1 | 0 | 0 | 0 | 0 | 7 | 0 |
|  | Mutant allele % | - | - | - | - | 4.8% | - | - | - | - | 8.8% | 1.0% |
